# Dual Functionality of Biobased Bacterial Nanocellulose as Polymer Additive for Drilling Operations and Post-Drilling Filter Cake Removal

**DOI:** 10.1021/acsomega.6c01413

**Published:** 2026-05-25

**Authors:** María Eugenia Taverna, Yurany Villada, Sebastián Dell Elce, Jimena Bovi, Carlos Busatto, Juan Martin Maffi, Camilo A. Franco, María Laura Foresti, Farid B. Cortés, Diana Estenoz

**Affiliations:** † INTEC (UNL-CONICET), Güemes 3450, Santa Fe S3000GLN, Argentina; ‡ UTN Facultad Regional San Francisco, San Francisco, Córdoba X2400ARJ, Argentina; § Grupo de Investigación Fenómenos de Superficie Michael Polanyi, Facultad de Minas, 28021 Universidad Nacional de Colombia, Medellín 050034, Colombia; ∥ ITPN (UBA-CONICET), Ciudad Autónoma de Buenos Aires C1428EGA, Argentina; ⊥ 28169 Instituto Tecnológico de Buenos Aires (ITBA), Lavardén 315, Ciudad Autónoma de Buenos Aires C1437FBG, Argentina; # Facultad de Ingeniería Química, Santiago del Estero 2654, Santa Fe S3000AOM, Argentina

## Abstract

This work explores bacterial nanocellulose (BNC) as a biobased and sustainable polymeric additive for water-based drilling fluids (WBMs). BNC was isolated from the cellulosic byproduct of kombucha tea production, yielding highly pure cellulose with high crystallinity and mechanical stability. WBMs prepared with BNC exhibited higher viscosities and similar pseudoplastic behavior compared with those formulated using xanthan gum (XGD). Thermal aging at 90 °C showed that BNC-based fluids retained around 80% of their initial viscosity, confirming superior thermal stability and longer service life. Although slightly higher filtrate volumes were recorded, the overall rheological performance was enhanced. Additionally, enzymatic treatment of BNC enables controlled viscosity reduction, which may facilitate fluid management during cementing operations. This dual functionality—high performance during drilling coupled with controlled postuse degradability—supports a circular materials strategy by reducing polymer consumption and minimizing waste generation. In this context, the proposed approach contributes to sustainable development goal (SDG) 12: Responsible Consumption and Production and SDG 9: Industry, Innovation and Infrastructure, through the development of sustainable materials for industrial applications. Overall, these results highlight BNC as a promising multifunctional polymer and provide initial insights for the development of alternative sustainable drilling fluid formulations.

## Graphical abstract

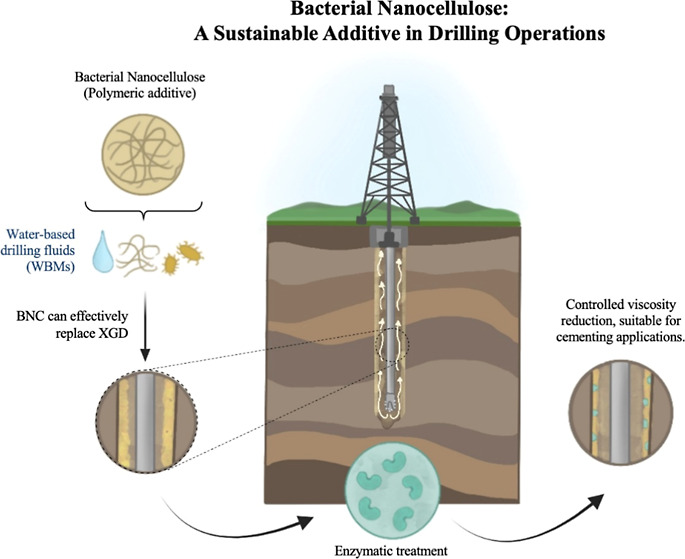

## Introduction

Drilling fluids play a crucial role in oil and gas drilling operations. They are essential for effective transporting of cuttings to the surface, maintaining desired rheological properties, cooling and lubricating the drilling bit, stabilizing the wellbore, and preventing damage to the formation.^1,2^ Drilling fluids can be classified into three categories: water-based drilling fluids (WBMs), oil-based drilling fluids (OBMs), and synthetic drilling fluids (SBMs).^3^ OBMs and SBMs present certain drawbacks, including health risks, environmental pollution, and high costs, which limit their widespread use.^4,5^ In comparison, WBMs are used due to their environmentally friendly characteristics. They consist of water, bentonite clay, and various additives such as weighting materials, thickeners, filtration control agents, deflocculants, and heat/salt-resistant agents. Bentonite (BT) clay, a fundamental component of WBMs, is particularly valuable for controlling the properties of the drilling fluid due to its excellent swelling capability and superior rheological properties.^2^

In conventional WBMs, bentonite (BT) clay acts as the primary structural component, providing viscosity and suspension capacity due to its swelling capability. Polymeric additives are also incorporated to tailor specific properties of the fluid.^6–9^ In particular, xanthan gum (XGD) is commonly used as a rheology modifier to enhance viscosity and shear-thinning behavior, while polyanionic cellulose (PAC) is typically employed as a filtration control agent to reduce fluid loss and improve wellbore stability.^10–13^ These components work together to ensure the appropriate rheological and filtration properties required for efficient drilling operations.^14^

Besides, in the context of actions devoted to the development of sustainable drilling materials, alternative polymer additives aligned with sustainable and circular practices such as guar gum^15,16^ and cellulose derivatives^4,15,17,18^ have also received great attention. On the other hand, there is a growing interest in using biobased micro/nanosized materials to enhance the performance of drilling fluids while minimizing their negative impact on the environment and human health.^19^ In this context, nanocellulose has demonstrated immense potential as an additive in water-based muds (WBMs), offering eco-friendly and high-performance solutions. Nanocellulose can be classified into three categories^20^ based on its preparation method: cellulose nanocrystals (CNCs), usually produced by acid hydrolysis of cellulosic raw materials; cellulose nanofibers (CNFs), obtained by physical and mechanical deconstruction processes; and bacterial nanocellulose (BNC), produced through microbial routes. Unlike CNF and CNC materials derived from lignocellulosic biomass through mechanical or chemical treatments, BNC is produced via microbial synthesis, yielding highly pure cellulose -free of lignin and hemicellulose- with a well-organized nanofibrillar structure improving with high crystallinity.^21,22^

Recent studies have advanced the understanding of nanocellulosic polymers from plant origin and their contribution to drilling fluid performance. Khan et al. (2024)^23^ presented a comprehensive review on celluloses, including CNFs and CNCs, highlighting their efficiency in sealing shale nanopores, preventing water intrusion, and improving fluid loss control.^24–26^ Mianehrow et al. (2022)^27^ demonstrated that cellulose nanomaterials enhance the dynamic performance of WBMs under operational stresses, while Koo et al. (2021)^28^ reported that CNF–XGD composites increase gel strength, viscosity, and dynamic modulus, mitigating the adverse effects of lignin. Our previous work showed that WBMs formulated with bleached and unbleached CNFs containing lignin exhibit superior thermal stability, suitable for high-temperature drilling environments.^17^ Guo et al. (2023)^2^ and Zhou et al. (2023)^29^ investigated the influence of CNF concentration, morphology, and surface chemistry on rheological and filtration properties, identifying CNFs with optimized aspect ratios and functional groups as potential XGD substitutes. Liu et al. (2024)^30^ developed modified CNCs as stabilizers for reversible emulsion drilling fluids, achieving enhanced thermal resistance (up to 150 °C), stability, and reduced surfactant demand. Khan et al. (2025)^31^ compared TEMPO-oxidized (T-CNFs) and mechanically disintegrated CNFs (M-CNFs) in bentonite-based WBMs under high-temperature, high-salinity conditions. T-CNFs improved salt tolerance and fluid loss control, whereas M-CNFs enhanced rheology and thermal resistance. These studies highlight the use of nanocellulose from plants as an advanced polymer material to control the structure and properties of drilling fluids.

In contrast to the extensive research devoted to plant-derived nanocelluloses, the application of BNC in water-based drilling fluids remains largely unexplored despite its more sustainable production and distinguishing characteristics. Bacteria produce cellulose with high purity, so the harsh chemical extraction steps required to remove lignin and hemicelluloses in plant-based cellulose are avoided.^20,32,33^ Besides, microorganisms already produce BNC in the form of entangled high-aspect ratio nanofibrils, so no high energy-demanding mechanical treatments or chemical processes that introduce new charged groups are required for isolation of nanoscale elements. Furthermore, BNC is distinguished for a highly crystalline structure which contributes to high mechanical and thermal properties. Accordingly, BNC is expected to be particularly beneficial in complex aqueous systems like water-based drilling fluids; as a result of reduced expected interference with other additives and more consistent and predictable rheological behavior derived from the absence of impurities; efficient rheological structuring derived from a highly homogeneous nanofibrillar network, and high thermal and chemical stability (unlike sulfate-functionalized CNCs, BNC does not carry thermally or ionically labile groups). Besides, the homogeneous three-dimensional nanofibrillar architecture and high surface area of BNC^34,35^ are particularly favorable for strong and consistent interactions with bentonite particles and other additives of water-based drilling fluids,^2,17,36^ superior dispersion stability and improved rheological control. Furthermore, cost-effective renewable sources of BNC are available; as it is the case of BNC isolation from Kombucha-derived pellicles which allow for valorization of a residue of an expanding beverage industry obtained under much less demanding conditions than single strain production of BNC.^37^ All these features trigger the interest for BNC as a promising alternative to plant-derived nanocelluloses for advanced drilling fluid formulations.^5,10,11^ In particular, BNC isolated from Kombucha-derived pellicles represents a promising alternative to conventional rheology modifiers such as XGD. Although XGD is widely used as an effective rheology modifier in water-based drilling fluids due to its shear-thinning behavior and ease of dispersion, its performance may be limited under demanding operational conditions such as high salinity, elevated temperatures, or prolonged mechanical stress. In this context, BNC, with recognized thermal stability and stable rheological behavior over time, can contribute to enhanced fluid stability and consistent performance during extended use. These features are particularly relevant in drilling operations involving fluid recirculation, where maintaining viscosity and suspension capacity is critical.

On the other hand, during drilling operation, and to ensure wellbore integrity after the removal of drilling fluids, an annular cement barrier is strategically placed between the steel casing and the surrounding rock formation. This barrier plays a crucial role in stabilizing the wellbore and preventing fluid migration.^38^ However, if the drilling fluid has a high viscosity, a leaching of the clay cake is created that hinders adhesion, therefore the cement does not cling to the rock. There are some treatments to partially remove the clay cake based on the reduction of the viscosity of drilling fluids. In the last years, several agents such as hydrochloric acid, weak organic acids, enzymes, chelating agents, and oxidizing agents have been utilized for the removal of filter cakes.^39–41^ Among these methods, enzymatic treatment avoids secondary damage to casings, tools, or surrounding rock, as enzymes do not interact with metals or formation rocks.^40^ In addition, the enzymatic treatment of filter cakes is slower compared to oxides and acids, ensuring a uniform removal. The activity of enzymes depends on the temperature, ionic conditions and the pH of the environment. Therefore, enzyme activity should be evaluated to determine the optimal conditions. Finally, enzymatic removal methods further align with the low-impact and selective degradation strategies valued in sustainable polymer engineering.

Although research on nanocellulose-based polymeric additives has advanced considerably, our understanding of how BNC behaves within the complex environment of drilling fluids remains surprisingly limited. The field has largely centered on vegetal-derived CNCs and CNFs, which has left important questions unresolved regarding the rheological response, thermal stability, and inhibition capacity of BNC under conditions relevant to drilling. Even more overlooked is the possibility of extending the role of BNC beyond the drilling stage itself. Little attention has been given to how this biopolymer might interact with postdrilling operations—particularly cementing—where its tunable degradability could support more sustainable fluid management strategies.

In this work, BNC is investigated as a biobased alternative to XGD in water-based drilling fluids. This work proposes an integrated approach combining the use of BNC as a drilling fluid additive with its controlled enzymatic degradation to facilitate postdrilling operations. This dual approach not only enhances the sustainability of the drilling process but also provides a potential solution to the challenges of clay cake removal and fluid compatibility during cementing. Drilling fluids based on BNC are designed and assessed in terms of rheological behavior, filtration, thermal stability and inhibition properties. In addition, the reutilization of fluids with BNC in the cementing operation is also addressed. To this effect, enzymatic treatment of WBMs is carried out by using cellulase at different concentrations and under different conditions of temperature and pH. By integrating biobased polymer design with strategies for controlled degradation, this work contributes to the development of more sustainable drilling fluid formulations and aligns with Sustainable Development Goals related to responsible consumption and industrial innovation.

## Materials and Methods

### BT, PAC, and XGD Characteristics

Bentonite (BT), polyanionic cellulose (PAC), and xanthan gum (XGD) were supplied by MARBAR S.R.L. (Argentina), M-I SWACO (Argentina), and Química Oeste S.A. (Argentina), respectively. PAC and XGD were used as received and correspond to industrial-grade products commonly used as rheological additives in drilling fluids. Bentonite corresponded to a sodium bentonite mainly composed of smectite.

Cellulase (Celluclast 1.5 L) was supplied by Novozymes (Denmark), with an enzymatic activity of 70 FPU mL^–1^.

According to our previous work,^11^ PAC exhibited a weight-average molecular weight (Mw) of 1,146,000 g mol^–1^, a polydispersity index (PDI) of 1.65, and a zeta (ζ) potential of −48.70 ± 0.5 mV under neutral conditions. XGD presented weight- and number-average molecular weights (*M*_w_ and *M*_n_) of 1,622,000 g mol^–1^ and 881,000 g mol^–1^, respectively, with a ζ potential of −34.3 mV.

Regarding BT, the material consisted mainly of smectite, with impurities such as quartz, feldspar, and gypsum. The FTIR spectrum showed an Al–Al–OH stretching vibration at 3623 cm^–1^, characteristic of smectites with a high aluminum content in the octahedral layer. The bands at 3447 cm^–1^ and 1644 cm^–1^ correspond to the stretching and bending vibrations of adsorbed water (H–O–H), respectively. The sharp peak at 1059 cm^–1^ was attributed to Si–O stretching vibrations. Tetrahedral bending modes were observed at 514 cm^–1^ (Si–O–Al) and 454 cm^–1^ (Si–O–Si). The OH bending vibration assigned to Al–Al–OH in dioctahedral 2:1 layer silicates appeared at 913 cm^–1^. Finally, peaks associated with quartz were observed near 779 cm^–1^ and 795 cm^–1^.^4^

### Production and Characterization of BNC

BNC was isolated from the floating pellicle formed at the air–liquid interface during the production of Kombucha tea under specific conditions (commercial black tea infusion 10 g/L, glucose 60 g/L, starter culture 10% v/v, static conditions, 28–30 °C, 14 days) due to the activity of acetic acid bacteria found in the fermented tea. The use of kombucha-derived pellicles provides a highly renewable and low-impact source of nanocellulose, consistent with sustainable polymer design principles. The harvested pellicles were purified with KOH, which included thorough washing with tap water at room temperature, grinding in KOH 0.9 M for 4 min using a kitchen blender, storage in alkali in static conditions for 14 h at room temperature, and thorough rinsing with distilled water until neutral pH.^35^

Nanocellulose morphology was characterized through field-emission scanning electron microscopy (FESEM) and transmission electron microscopy (TEM). Microstructure was analyzed through optical microscopy using a Leica optical microscope under transmitted light. FESEM micrographs of BNC were obtained using a field-emission scanning electron microscope (FEI Quanta 200) operated at an acceleration voltage of 15 kV. Samples were deposited on microscopy glass slides as aqueous suspensions (∼0.1% w/v), dried at 110 °C for 1 h, and sputter-coated prior to observation. TEM micrographs of BNC were obtained using a TEM Philips EM 301 microscope operated at an accelerating voltage of 40 kV. Samples were negatively stained with 2 wt % uranylacetate.

The thermal stability of BNC was determined by thermogravimetric analyses (TGA). To this effect, a TA Instruments Q500 thermobalance was utilized. Using a platinum pan, the heating rate was 10 °C/min, increasing temperature from room temperature up to 800 °C in a nitrogen environment (100 mL/min). The sample size was about 5 mg of dry sample. The onset degradation temperature was determined using the tangent method, as recommended in the literature^42^

The FTIR spectrum was recorded using a Shimadzu FTIR-8201PC spectrophotometer. A KBr pellet was prepared with 2–3%wt. of dry sample and it was measured in the frequency range of 4000–400 cm^–1^. The characteristic signals were assigned according to Morais et al.^43^

A Zetasizer Nanoseries Malvern (ZS90) was utilized to determine net surface charge by measuring the Zeta potential (ζ) of a BNC neutral suspension (1 g/L).

The X-ray diffraction pattern (XRD) of the samples was scanned on a Shimadzu XD-D1 diffractometer in a 2θ range between 10° and 40°, using Cu–Kα radiation (λ = 0.1541 nm, 2°/min, 40 kV and 30 mA). The crystallinity index (CrI) of BNC was determined using the Segal^44^ and deconvolution methods as described by Park et al.^45^

The average viscometric degree of polymerization (DPv) was determined by viscometry of a lyophilized sample of BNC. The sample was dissolved in a 0.5 M cupriethylenediamine (CUEN) solution, and the specific viscosity was measured at 20 °C. Then the intrinsic viscosity (η) was obtained and the DPv was determined. It is important to not that all the characterization techniques, including FESEM, TEM, FTIR, XRD, TGA, and ζ potential measurements, were conducted using standard instrumental protocols commonly reported in the literature for nanocellulose materials.

### Inhibition Test for XGD and BNC Suspensions

The linear swelling test of BT was performed to evaluate the inhibition properties of the XGD and BNC suspensions. The BT pellet, obtained by pressing the clay for 5 min under a pressure of 86 MPa, was used to assess the inhibition efficiency by measuring its linear vertical expansion (linear swelling rate, %) at different time intervals (5, 15, and 30 min; 1, 4, and 6 h) in 1 wt % XGD or BNC suspensions.^46^ The measurements were carried out in quintuplicate.

### WBMs Preparation

Two simple water-based mud (WBM) systems were designed: BNC/BT/PAC/H_2_O and XGD/BT/PAC/H_2_O. The compositions of the formulations are presented in Table 1. Each fluid was coded according to its BT content and the type and concentration of the structuring additive, while the concentration of PAC was kept constant in all formulations. In the codes, BT1 and BT4.5 denote formulations containing 1.00 and 4.50 wt % BT, respectively. The term Base refers to the reference fluid without BNC or XGD, containing only bentonite, PAC, and water. The terms BNCx and XGDx indicate the addition of bacterial nanocellulose or xanthan gum at concentration x (wt %).

**Table 1:** Composition of Simple Water-Based Muds (wt %)^a^

**fluid**	BT **(wt %)**	PAC **(wt %)**	**additive**	conc. **(wt %)**
**BT1-base**	1.00	0.50	—	0.00
**BT1-BNC0.10**	1.00	0.50	BNC	0.10
**BT1-BNC0.25**	1.00	0.50	BNC	0.25
**BT1-BNC0.50**	1.00	0.50	BNC	0.50
**BT1-XGD0.10**	1.00	0.50	XGD	0.10
**BT1-XGD0.25**	1.00	0.50	XGD	0.25
**BT1-XGD0.50**	1.00	0.50	XGD	0.50
**BT4.5-base**	4.50	0.50	—	0.00
**BT4.5-BNC0.10**	4.50	0.50	BNC	0.10
**BT4.5-BNC0.25**	4.50	0.50	BNC	0.25
**BT4.5-BNC0.50**	4.50	0.50	BNC	0.50
**BT4.5-XGD0.10**	4.50	0.50	XGD	0.10
**BT4.5-XGD0.25**	4.50	0.50	XGD	0.25
**BT4.5-XGD0.50**	4.50	0.50	XGD	0.50

a BNC: bacterial nanocellulose; BT: bentonite; PAC: polyanionic cellulose; XGD: xanthan gum.

The fluid preparation followed the Standard Procedure for Testing Drilling Fluids outlined in the API Recommended Practice 13B-1 (2003). Initially, BT was hydrated at room temperature for 16 h. Subsequently, a suspension of BNC or XGD was introduced. Finally, PAC was added, and the mixture was stirred thoroughly until a homogeneous consistency was achieved.

### WBMs Characterization

The performance of WBMs was characterized by considering the thermal and physicochemical properties of all polymeric additives, the interactions that influence overall system behavior.

The rheometric properties of the drilling fluid were measured by using a Brookfield DV3TRV viscometer. The cone–plate CP-51Z configuration was used with a shear rate range of 3.84–960 1/s at 25 °C. For each measurement, 3 replicates were performed to guarantee the reproducibility of the results. Additionally, plastic viscosity (PV) and yield point (YP) were estimated using the Bingham plastic model from the linear region at high shear rates (512–1022 s^–1^), according to API recommendations.

Viscosity retention (%) was calculated as the ratio between the viscosity measured at the highest and lowest shear rates (ηhigh/ηlow × 100). Statistical comparisons between BNC and XGD systems were performed using a *t*-test with a significance level of *p* < 0.05.

The Power Law rheological model was used to fit the experimental data

1 $$\eta =k{\mathrm{\gamma ̇}}^{n-1}$$

where *k* (mPa.s) is the consistency index, γ̇ (1/s) is the shear rate and *n* is the flow behavior index.

In addition, the Herschel–Bulkley model was applied to account for the presence of yield stress

2 $$\tau =\tau_{0}+K{\mathrm{\gamma ̇}}^{n}$$

where τ (Pa) is the shear stress, τ_0_ (Pa) is the yield stress, *K*(Pa·s^*n*^) is the consistency index, and *n* is the flow behavior index.

### Dynamic-Aging Test

This test provides a direct assessment of the thermal resilience of the polymer network.

To assess the thermal stability of the WBMs, selected fluids were exposed to a temperature of 90 °C for 24 h. After this period, the rheometric tests were conducted again following the previously outlined procedure.

### Structural Properties

Scanning electron microscopy (SEM) was used to analyze the microstructure of simple fluids. For SEM analysis, the samples covered with gold were examined using a acceleration voltage of 5 keV at a working distance of approximately 4.5 mm, using a Carl Zeiss Supra 35VP microscope (Germany).

### Filtration Properties

Filtration tests were carried out according to the API standards (API recommended practice 13B-1, 2003). To this effect, a filter press was used with CO_2_ as pressurizing gas at 100 psi and with No. 50 Whatman filter paper. Filtrate readings were performed at 5, 7.5, 10, 15, 20, 25, and 30 min at room temperature. First reading was considered to determine the instantaneous filtrate. In addition, aged fluids were filtrated to evaluate the effect of aging. The test was conducted in triplicate.

### Enzymatic Treatment

This experimental protocol was designed to evaluate the selective degradability of BNC as a biobased polymer, a key factor in enabling circularity within the fluid system. The efficiency of polymer degradation by the enzymatic treatment of WBMs was determined based on the modifications of the rheological properties of the drilling muds following the introduction of varying enzyme concentrations (0.1% and 0.3%) at different temperatures (20 and 50 °C) and pH conditions (5 and 9). A 2-level factorial design (2^3^) was employed to identify the parameters that have a more preponderant effect on the rheological properties of WBMs. The response variable considered in the experimental design was the viscosity measured at a shear rate of 576 s^–1^. The factorial design allows for the identification of the conditions under which the enzymatic breakdown of the polymer becomes functionally relevant for viscosity reduction. The reproducibility of the experimental results within the RSM design was assessed through replicated runs and statistical analysis of the fitted model.

Polymer degradation was evaluated indirectly through viscosity changes, since the drilling fluid is a multicomponent system in which direct determination of polymer degree of polymerization is difficult.

## Results and Discussion

### BNC Characteristics

The ζ potential of the BNC was determined to be −9.79 ± 1.68 mV. It is noteworthy that similar values have been reported for other types of nanocellulose and are commonly attributed to the presence of surface hydroxyl groups.^47,48^ The ζ potential value of −9.79 mV indicates a slight negative surface charge on BNC, which may contribute to its interactions with other components in the aqueous drilling fluid system. The micrographs of BNC obtained by field emission scanning electron microscopy (FESEM) and transmission electron microscopy (TEM) are presented in Figure 1a,b, respectively. The dimensions of the nanofibrils were determined from the micrographs using ImageJ software (version 1.53e). Optical microscopy images are depicted in Figure 1c, revealing the presence of nanofiber agglomerates, which is commonly observed in this type of nanocellulose materials.

**Figure 1:**
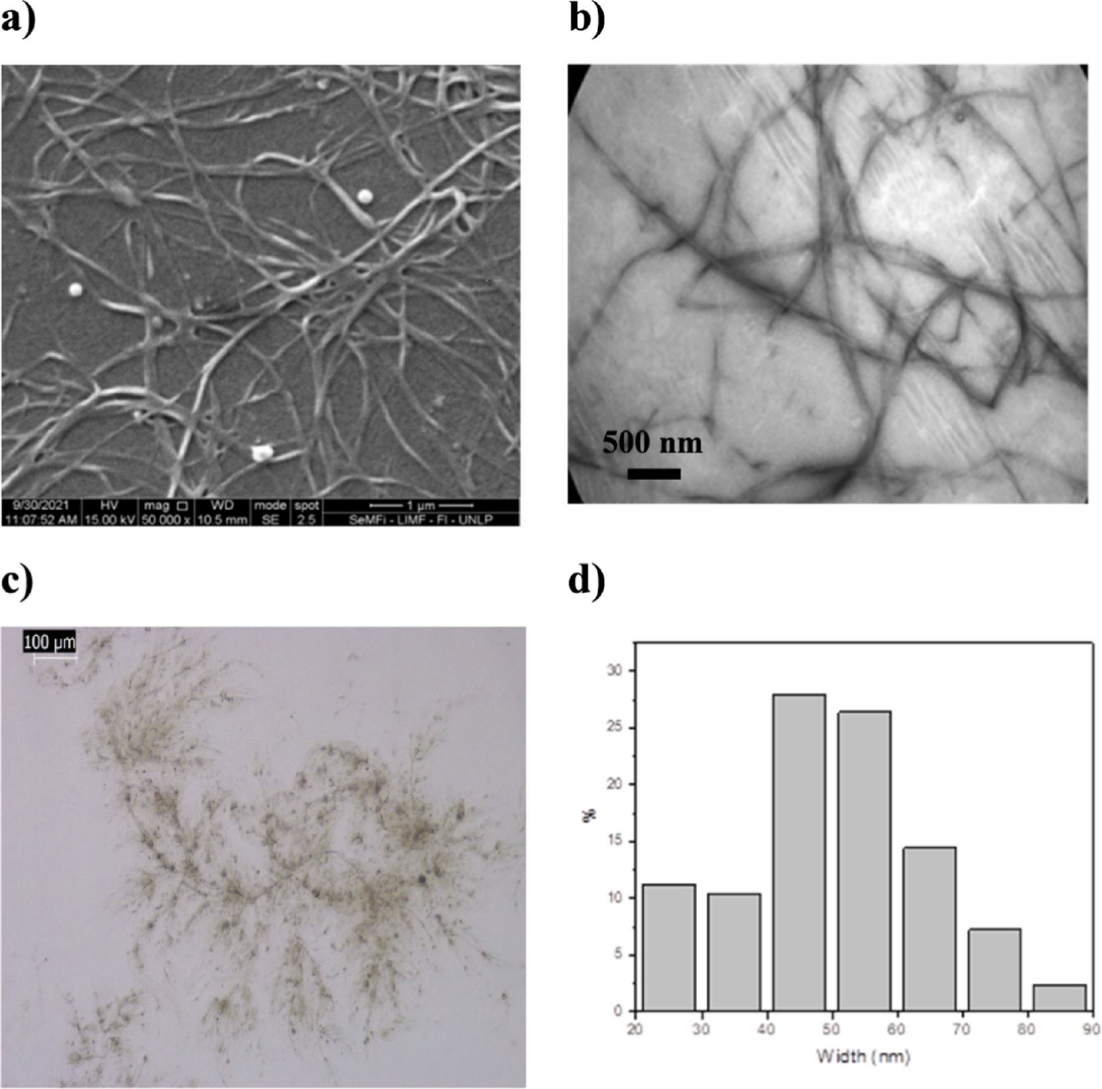
Morphological structure of BNC observed by FESEM (a, scale = 1 μm), TEM (b, scale = 0.2 μm), optical microscopy (c, scale = 100 μm), and the corresponding histogram (d).

The FESEM/TEM micrographs included in Figure 1 show intricately intertwined nanofibrils with widths within the 20–90 nm interval, in agreement with the typical dimensions reported for BNC.^37,49^ Figure 1d shows the width distribution of the bacterial cellulose nanofibers used. In reference to nanofibers length, they are several micrometers long,^49^ exceeding the observation window of FESEM and TEM images.

The thermal behavior of BNC, shown in Figure 2a, indicates high thermal stability, with a maximum degradation temperature of approximately 370 °C. The char residue of 13% observed at the end of the analysis reflects the formation of a stable carbonaceous phase after thermal decomposition. These values are consistent with those reported in the literature for BNC.^34^ The FTIR spectrum of BNC (Figure 2b) exhibits the characteristic absorption bands of cellulose. The broad O–H stretching band around 3300–3400 cm^–1^ is characteristic of cellulose and is associated with the stretching vibration of −OH.

**Figure 2:**
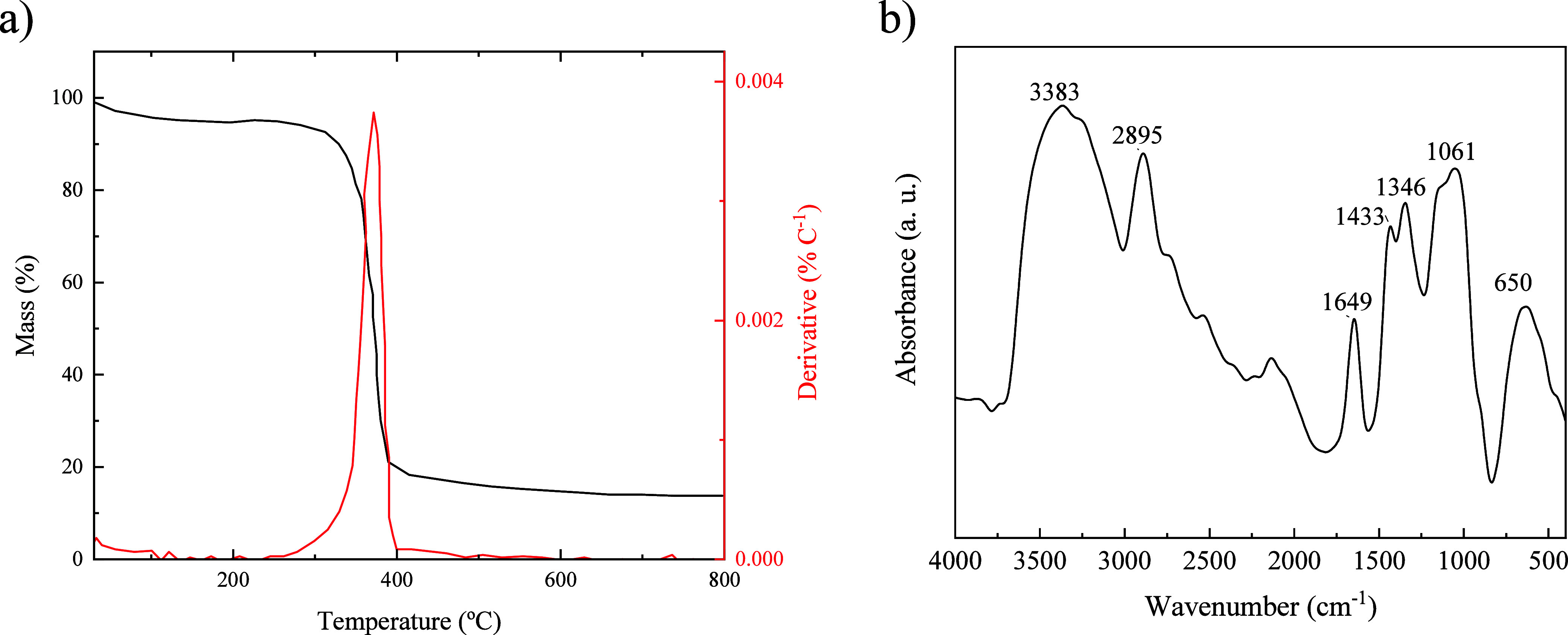
(a) TGA curve of BNC. (b) FTIR spectrum showing characteristic bands of cellulose.

The peak at 2895 cm^–1^ is attributed to C–H stretching. The band at 1649 cm^–1^ arises from O–H bending of adsorbed water. The peaks at 1433 cm^–1^ and 1346 cm^–1^ are assigned to −CH_2_ vibrations and to C–H and C–O bending in the polysaccharide structure, respectively.^50,51^ The intense band at 1061 cm^–1^ corresponds to C–O–C stretching of the pyranose ring, and the one at 650 cm^–1^ is related to δCOH out-of-plane bending.

The X-ray diffraction (XRD) pattern of the BNC aligns closely with the characteristic XRD of Cellulose I, as illustrated in Figure 3. This observation indicates a specific typical crystalline structure of cellulose, highlighting the ordered arrangement of the BNC molecular chains. The intensity of the 200 plane, as well as the intensity of the amorphous contribution recommended by Segal’s method were used to estimate the crystallinity index of BNC. A value of 93 ± 1% was calculated by this empirical method, in accordance with previous reports that used the same methodology for calculation of CrI.^49,52^

**Figure 3:**
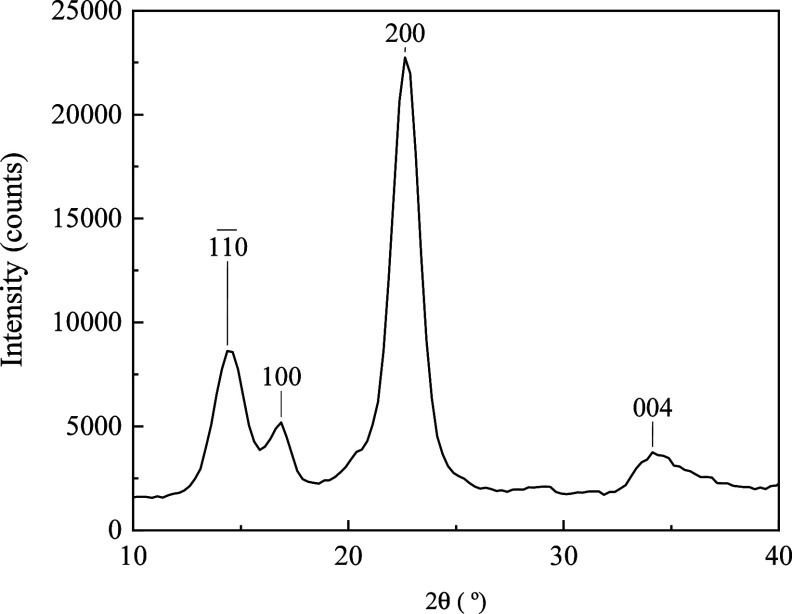
XRD patterns of BNC.

Besides, since Segal’s method is known to produce values that are significantly higher than the other methods,^45^ the CrI of BNC was also calculated by deconvolution using Gaussian functions. The CrI value of BNC determined by this method was 76 ± 1%, also in accordance with previous literature using the same methodology.^45,52^ The high crystallinity index values obtained by both methods are characteristic of BNC, indicating a well-organized and tightly packed cellulose structure which contributes to the material’s overall stability and mechanical strength.^44^

The average viscometric degree of polymerization (DPv) for BNC was determined to be 8181 ± 1583. This value falls within the typical range for BNC, indicating the average number of monomeric units (glucose molecules) in a polymer chain.^32^ Considering that the molecular weight of a monomeric unit of cellulose is 162.4 g/mol, the average molecular weight of the polymer is approximately 1.30 × 10^6^ g/mol.

Overall, the morphological, thermal, and structural characteristics of BNC make it a highly promising candidate for incorporation into fluid formulations. Its high crystallinity (90%), elevated degree of polymerization (DPv = 8181), and onset degradation temperature of 354 °C ensure mechanical and thermal resistance under demanding conditions. Moreover, the nanometric dimensions of the fibers and the negative ζ potential contribute to improve their dispersibility and stability in aqueous media, which are essential properties for its effective integration into fluid systems, such as drilling fluids or other advanced colloidal suspensions. These structural and molecular attributes directly influence the formation, rigidity, and thermal resilience of the polymeric network formed within WBMs.

### Inhibition Assay

As shown in Figure 4, both XGD and BNC effectively inhibit the swelling of BT compared to its behavior in water. The inhibition test was performed using polymer suspensions (BNC and XGD) without the complete drilling fluid formulation, in order to isolate the specific effect of each additive on clay hydration. When full drilling fluid systems are used, the presence of dispersed BT particles and polymer networks leads to opaque and structurally complex systems, which hinder reliable visualization and measurement of pellet expansion. Therefore, the use of polymer suspoensions in inhibition assays is a commonly adopted approach to ensure accurate and reproducible evaluation of swelling behavior. The inhibition is more pronounced in the presence of XGD, which can be attributed to its more negative ζ potential (≈− 34 mV) relative to BNC (≈− 10 mV). The higher anionic charge density of XGD promotes compression of BT’s electrical double layer and counterion accumulation, thereby reducing the osmotic component of swelling. Additionally, XGD adsorption could occur on positively charged edge sites and the formation of a viscous steric network restricts interlayer expansion and enhances colloidal stability. This behavior agrees with previous reports describing anionic biopolymers that inhibit clay hydration through combined electrostatic and steric mechanisms.^53,54^ In contrast, BNC exhibits a significantly lower ζ-potential (−10 mV) compared to XGD (−34 mV). Consequently, its interaction with BT is mainly governed by hydrogen bonding and physical confinement, with minimal electrostatic contribution, in agreement with previous studies on the colloidal behavior of nanocellulose.^55^

**Figure 4:**
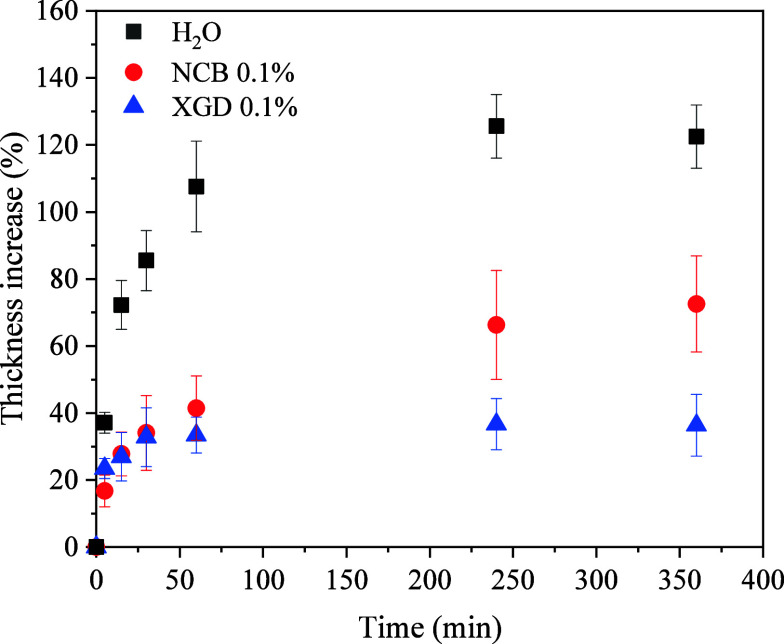
Inhibition assay of BT in H_2_O and in BNC and XGD polymer suspensions. The test consists of measuring the time-dependent swelling of a compacted bentonite pellet by monitoring its thickness increase.

### WBMs Characterization

Figure 5 shows the SEM micrographs of BT1-XGD0.50, BT1-BNC0.50, BT4.5-XGD0.50, BT4.5-BNC0.50 fluids. The incorporation of the biopolymers markedly altered the microstructure of the corresponding fluids. When comparing samples at equal BT content, BT1-XGD0.50 shows larger and more compact flocs than BT1-BNC0.50, which displays a relatively smoother and more homogeneous matrix. This observation is consistent with the more negative ζ potential of XGD, leading to electrical double-layer compression and polymer bridging between clay lamellae. Such interactions can promote flocculation at the microscale, even though they reduce interlayer swelling, as previously discussed. This behavior agrees with reports describing that highly anionic polysaccharides can simultaneously inhibit hydration and enhance aggregation through charge screening and cation bridging^56^

**Figure 5:**
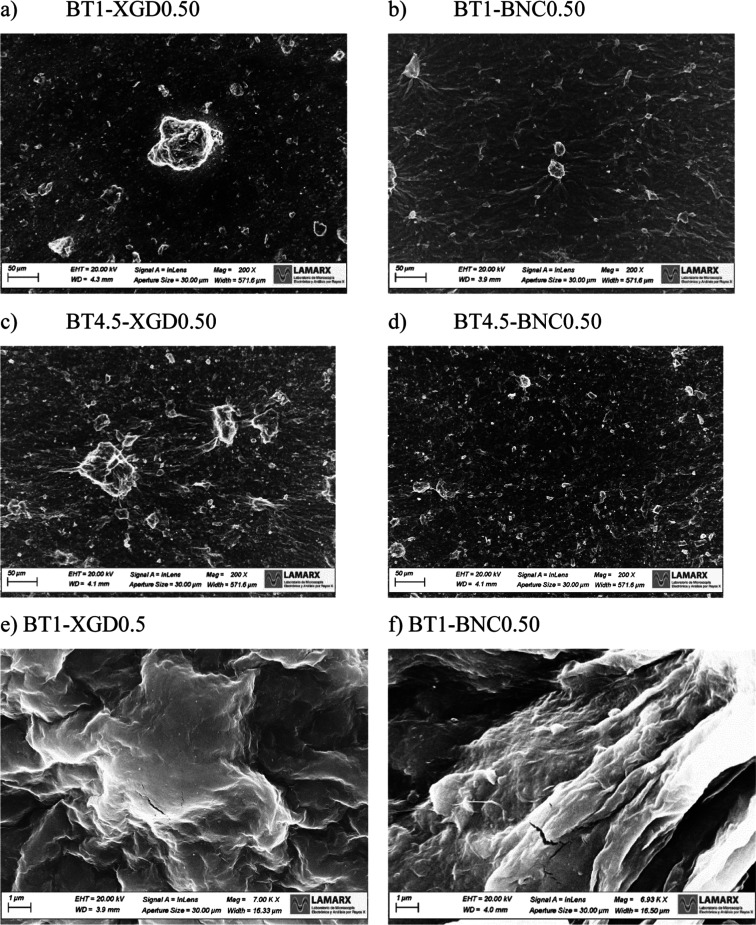
SEM micrographs of WBM fluids: (a) BT1-XGD0.50, (b) BT1-BNC0.50, (c) BT4.5-XGD0.50, and (d) BT4.5-BNC0.50, illustrating the effect of polymer type and bentonite content on particle aggregation and dispersion. High-magnification images of BT1-XGD0.50 (e) and BT1-BNC0.50 (f). Scale bar = 50 μm.

At higher BT content (BT4.5-XGD0.50 and BT4.5-BNC0.50), aggregation becomes more pronounced for both systems due to increased particle–particle contact and lower polymer coverage. However, BT4.5-BNC0.50 maintains a comparatively more open texture, suggesting that the physical entanglement and hydrogen bonding of BNC fibrils still limit particle coalescence. Although the fibers are not clearly resolved at this magnification, their presence likely contributes to a steric confinement effect. In contrast, XGD systems exhibit denser clusters, consistent with cation–exchange interactions between the anionic polymer and interlayer cations of BT, promoting localized floc formation.^57^

The higher-magnification micrographs shown in Figure 5e,f confirm the presence of BNC fibrils associated with the clay platelets. These structures provide direct evidence of the physical network responsible for steric stabilization, complementing the morphological differences observed in Figure 5a,b,c,d. The fibrous arrangement surrounding BT particles explains the reduced aggregation and smoother texture of BNC-containing fluids, in contrast to the electrostatically driven flocculation seen in XGD systems.

The rheological behavior of the formulated fluids is presented in Figure 6. All fluids exhibited high viscosities at low shear rates and pronounced shear-thinning behavior, characteristic of structured and pseudoplastic systems. The incorporation of the biopolymers promoted the formation of internal networks within the BT–PAC dispersions, increasing resistance to flow at rest and facilitating deformation under shear.

**Figure 6:**
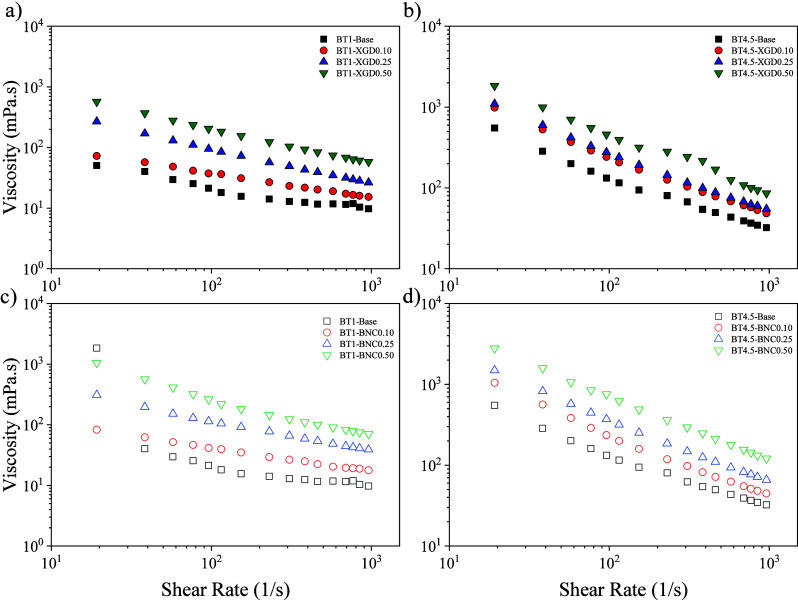
Rheological behavior of WBM systems containing XGD (a,b) and BNC (c,d) at different bentonite concentrations. Fluid codes correspond to Table 1.

In the case of XGD-containing fluids, the observed viscosities can be attributed to the entanglement of long and flexible polymer chains together with electrostatic interactions between the anionic polysaccharide and BT surfaces. This interpretation is consistent with the commonly reported rheological behavior for charged polysaccharides in aqueous suspension, where both physical entanglements and electrostatic interactions contribute to the overall viscoelastic response.^58^ The concentration range tested (0.25–1% w/v) ensures stable flow behavior without gelation, as polymer chains remain partially isolated and do not form a continuous network.^59^

In order to quantitatively compare the resistance of the fluid structure to shear under nonaged conditions, the viscosity retention percentage was calculated as the ratio between the viscosity at the highest and lowest shear rates. All formulations exhibited low viscosity retention values due to their pronounced shear-thinning behavior. Under these conditions, XGD-based systems showed a higher average viscosity retention (0.61%) compared with BNC systems (0.31%); however, statistical analysis (*t*-test, *p* = 0.43) indicated that these differences were not globally significant. Importantly, the lower viscosity retention observed in BNC systems reflects a more pronounced shear-thinning behavior, which is advantageous in drilling operations, as it facilitates pumping at high shear rates while maintaining high viscosity at low shear conditions.

By contrast, BNC-containing fluids displayed even higher apparent viscosities for equivalent BT contents. At a shear rate of 96 s^–1^, fluid BT1-BNC0.50 exhibited 263 mPa·s compared with 205 mPa·s for BT1-XGD0.50, while BT4.5-BNC0.50 reached 756 mPa·s versus 458 mPa·s for BT4.5-XGD0.50. This enhanced rheological response correlates with the fibrous microstructure observed in the SEM analysis and can be attributed to entanglement and hydrogen-bonding interactions between BNC fibrils and BT platelets, which generate a percolated network that resists deformation under low shear conditions. Consequently, at a given shear rate, the shear stress of BNC-containing fluids is higher than that of XGD-based systems due to the formation of a fibrillar network between BNC and bentonite particles.

Interestingly, this occurs despite the lower swelling and reduced aggregation found in BNC systems. This apparently opposite behavior arises from the coexistence of two counteracting effects: (i) limited BT hydration reduces the contribution of lamellar expansion to viscosity, but (ii) the formation of a cohesive fibrillar network compensates for this reduction, conferring greater structural integrity and resistance to flow. Similar effects have been reported for CNF–clay composites, where nanofiber-induced structuring dominates the rheological response over particle hydration.^60,61^

Consequently, the viscosity enhancement in BNC fluids arises primarily from physical network formation rather than electrostatic thickening, consistent with the steric stabilization observed in micrographs. The inherent crystallinity and rigidity of BNC further reinforce this network, explaining both the strong shear-thinning behavior and the higher consistency index (*k*) obtained from the Power Law model fits (Figure 7 and Table 2).

**Figure 7:**
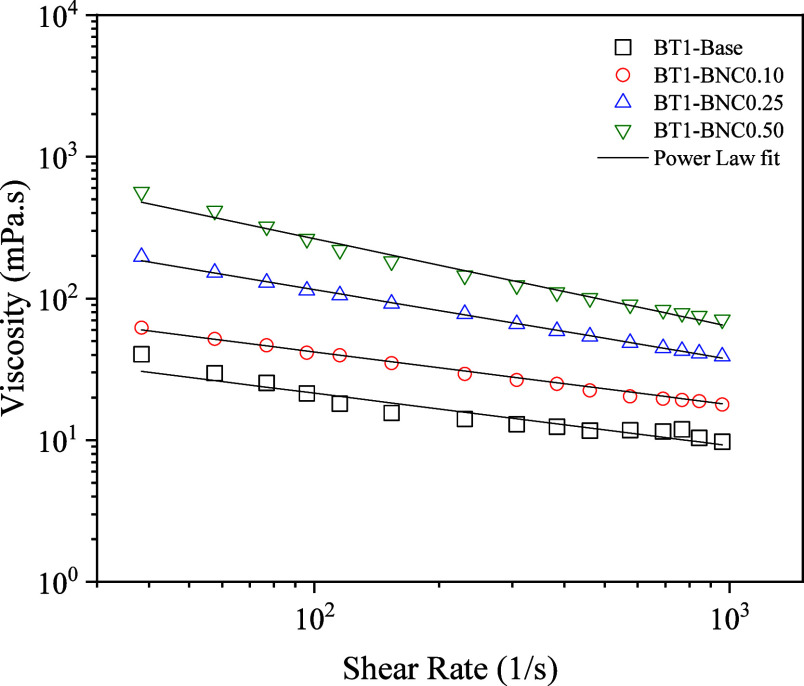
Power-law fitting curves for fluids BT1-Base, BT1-BNC0.10, BT1-BNC0.25, BT1-BNC0.50.

**Table 2:** Rheological Parameters Obtained from Power Law, Herschel–Bulkley and Bingham Models

	**power law**			**Herschel**–**Bulkley**				**Bingham**	
**fluid**	*k* (Pa·s^*n*^)	*n*	*R* ^2^	τ_0_ (Pa)	*K* (Pa·s^*n*^)	*n*	*R* ^2^	PV (Pa·s)	YP (Pa)
**BT1-base**	118	0.629	0.913	0.00	0.03	0.863	0.990	0.0065	3.37
**BT1-BNC0.10**	233	0.627	0.972	0.50	0.17	0.668	0.998	0.0119	5.67
**BT1-BNC0.25**	1107	0.509	0.995	3.94	0.37	0.655	0.999	0.0244	14.02
**BT1-BNC0.50**	4571	0.381	0.980	12.86	0.65	0.646	0.993	0.0414	28.46
**BT1-XGD0.10**	243	0.595	0.998	0.23	0.20	0.625	0.999	0.0099	5.17
**BT1-XGD0.25**	1317	0.428	0.998	1.49	0.82	0.491	0.999	0.0138	12.19
**BT1-XGD0.50**	2783	0.431	0.998	3.60	1.58	0.507	0.999	0.0333	23.71
**BT4.5-base**	2691	0.351	0.988	0.00	0.31	0.643	0.988	0.0154	16.31
**BT4.5-BNC0.10**	8092	0.233	0.990	5.84	0.91	0.558	0.993	0.0181	25.45
**BT4.5-BNC0.25**	12986	0.224	0.997	9.76	1.28	0.521	0.996	0.0242	40.15
**BT4.5-BNC0.50**	26493	0.213	0.997	18.46	2.03	0.497	0.997	0.0336	83.73
**BT4.5-XGD0.10**	6650	0.279	0.994	3.22	0.85	0.575	0.994	0.0197	28.29
**BT4.5-XGD0.25**	7948	0.269	0.996	4.98	1.10	0.552	0.996	0.0235	30.40
**BT4.5-XGD0.50**	13690	0.268	0.991	8.74	1.65	0.539	0.995	0.0269	56.63

All fluids exhibited flow indices (*n*) below 1, confirming their pseudoplastic (shear-thinning) behavior. As the concentrations of BT and polymer increased, the consistency index (*k*) increased and the flow index (*n*) decreased, consistent with previous findings for polymer–clay and nanocellulose–clay dispersions.^55,62^ As shown in Table 2, increasing BNC concentration leads to a marked increase in *k* together with a decrease in the flow behavior index (*n*), indicating the development of a more structured and shear-thinning fluid. This effect is more pronounced in BNC-based fluids than in XGD systems at comparable concentrations, indicating a stronger network structuring capability of BNC. This behavior agrees with the results reported previously by^5,12–14,63^

The Herschel–Bulkley model provided additional insight into the structural organization of the fluids, particularly through the estimation of yield stress (τ_0_), which is a key parameter for drilling applications as it reflects the ability of the fluid to suspend cuttings under static conditions.

As shown in Table 2, BNC-based fluids exhibited significantly higher yield stress values compared to XGD systems at equivalent concentrations. For instance, τ_0_ increased from 0.500 Pa (BT1-BNC0.10) to 12.856 Pa (BT1-BNC0.50), while the corresponding increase for XGD systems was less pronounced (0.225 to 3.595 Pa).

This behavior indicates that BNC promotes the formation of a stronger interconnected network, which resists flow initiation and enhances structural stability at low shear conditions. Such behavior is consistent with the nanofibrillar morphology of BNC, where physical entanglement and hydrogen bonding between fibrils and clay platelets lead to the development of a percolated network.

In contrast, XGD-based systems rely primarily on electrostatic interactions and polymer chain entanglement, resulting in weaker yield stress development. Therefore, the Herschel–Bulkley analysis quantitatively supports the hypothesis that the superior rheological performance of BNC systems arises from a structurally reinforced network rather than purely electrostatic thickening mechanisms. In addition to the Herschel–Bulkley analysis, plastic viscosity (PV) and yield point (YP) were estimated using the Bingham plastic model to facilitate comparison with standard drilling fluid engineering parameters. As shown in Table 2, both PV and YP increased with polymer concentration, although the variation in YP was significantly more pronounced. This trend confirms that the incorporation of biopolymers primarily enhances the structural strength of the fluid rather than only increasing viscous dissipation. Consistent with the Herschel–Bulkley results, BNC-based systems exhibited higher YP values than XGD systems, indicating a greater resistance to flow initiation. However, it is important to note that YP represents an empirical parameter derived from high shear rate data, and therefore does not strictly correspond to the true yield stress (τ_0_) obtained from the Herschel–Bulkley model.

In Figure 8, viscosity curves for BT1-BNC0.50 and BT1-XGD0.50 (a), and BT4.5-BNC0.50 and BT4.5-XGD0.50 (b) fluids before and after aging tests at 90 °C are presented. The rheological properties for fluids containing XGD showed a decrease of viscosity due to thermal treatment which is attributed to the increment of Brownian motion, the reduction of hydrogen bonds, and to polymer degradation at the tested conditions. For fluids containing BNC, the thermal stability was higher than fluids containing XGD. Additionally, the preservation of viscosities after the aging process indicates a minor loss of gel structure, which increases the useful life of the fluid and decreases the amount of polymer required during the entire operation.^64^ Compared with XGD systems, BNC-based fluids exhibited a smaller viscosity reduction after thermal aging at 90 °C for 24 h, indicating greater thermal stability of the nanocellulose network. In contrast to the behavior observed under nonaged conditions, BNC systems retained a high fraction of their initial viscosity after aging. This result highlights the robustness of the fibrillar network formed by BNC, which appears to be less susceptible to thermal degradation than the flexible polymer chains of XGD. Consequently, while XGD systems show slightly higher viscosity retention under shear in nonaged conditions, BNC provides improved structural stability under thermal stress, which is particularly relevant for drilling operations at elevated temperatures.

**Figure 8:**
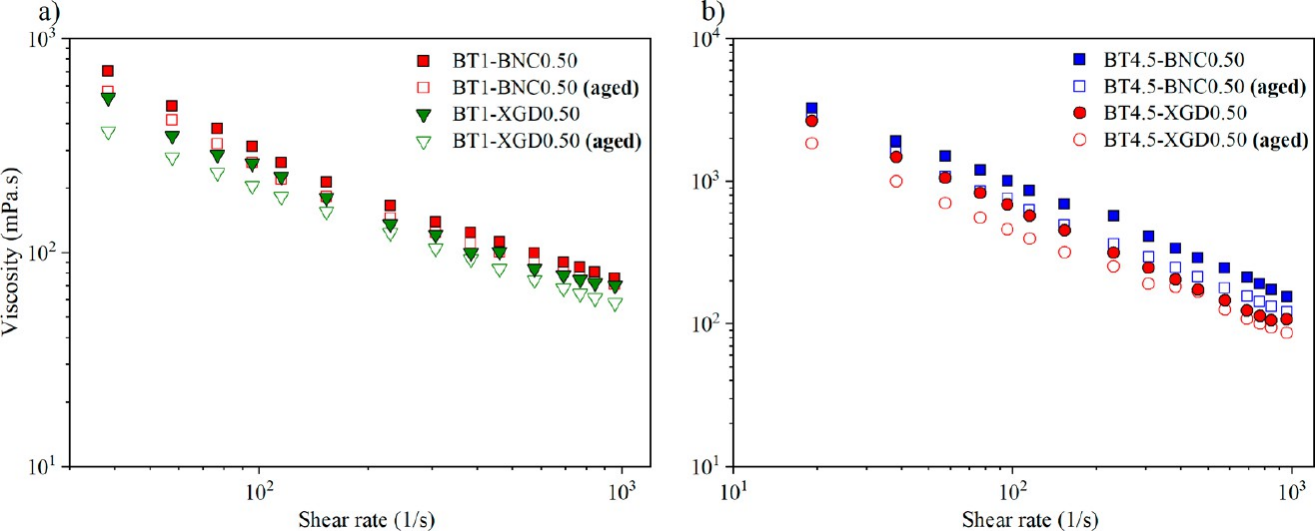
Rheological behavior of WBMs formulated with BNC and XGD before and after thermal aging at 90 °C for 24 h: (a) BT1-BNC0.50 and BT1-XGD0.50; (b) BT4.5-BNC0.50 and BT4.5-XGD0.50.

The filtration test results for BNC-containing fluids before and after aging are presented in Figure 9. In all cases, filtrate volumes increased after thermal aging, indicating partial degradation of the fluid microstructure. This effect was most pronounced in formulations with lower BT content, such as BT1-BNC0.50, which showed filtrate volumes at 30 min of approximately 13 mL before aging and 15 mL after aging. In contrast, BT4.5-BNC0.50 maintained the lowest filtrate volumes (around 7 mL after 30 min), demonstrating better structural stability and filtration control. A filtrate volume close to 7 mL is within the range reported for efficient water-based muds, as lower filtrate volumes generally indicate better filtration performance and the formation of a compact, low-permeability mud cake.^65,66^

**Figure 9:**
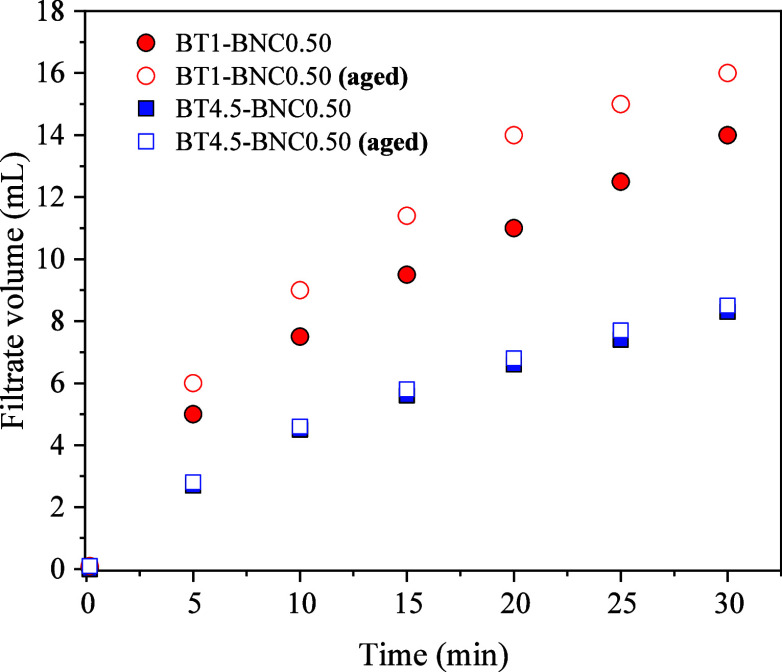
Filtration volumes of BT-based WBMs containing BNC before and after thermal aging, showing the effect of bentonite content on fluid loss.

For comparison, Villada et al.^17^ reported a filtrate volume of approximately 6 mL for the BT4.5-XGD0.50 fluid under similar test conditions. The slightly lower filtrate observed in XGD-based systems can be explained by the stronger and more homogeneous BT–XGD network formed through electrostatic and cation-bridging interactions between the anionic polysaccharide and the positively charged clay edge sites. These interactions promote particle flocculation and create a dense polymer–clay framework that retains water more effectively.^55^

### Enzymatic Treatment

The BCN-containing drilling fluids were enzymatically treated with cellulase under different experimental conditions. The results, shown in Figure 10, reveal that the viscosity of the drilling muds decreases markedly under acidic conditions, indicating a strong responsiveness of the system to pH during the enzymatic degradation of BCN. The enzymatic treatment resulted in a noticeable reduction in viscosity, indicating polymer degradation. The observed viscosity reduction corresponds to the laboratory conditions employed in this study. Under field-relevant conditions, the effective time scale may vary depending on factors such as temperature, enzyme concentration, and fluid composition. This behavior underscores the importance of maintaining precise pH control to optimize the effectiveness of the enzymatic breakdown process. The enzyme concentration also played a significant role in determining the rheological behavior of the fluids. Increasing the cellulase dosage accelerated the degradation of the cellulose component in the mud, resulting in a faster decrease in viscosity. This effect was clearly pH-dependent, as a noticeable viscosity reduction occurred only at pH 5, which corresponds to the optimum activity range of the enzyme.

**Figure 10:**
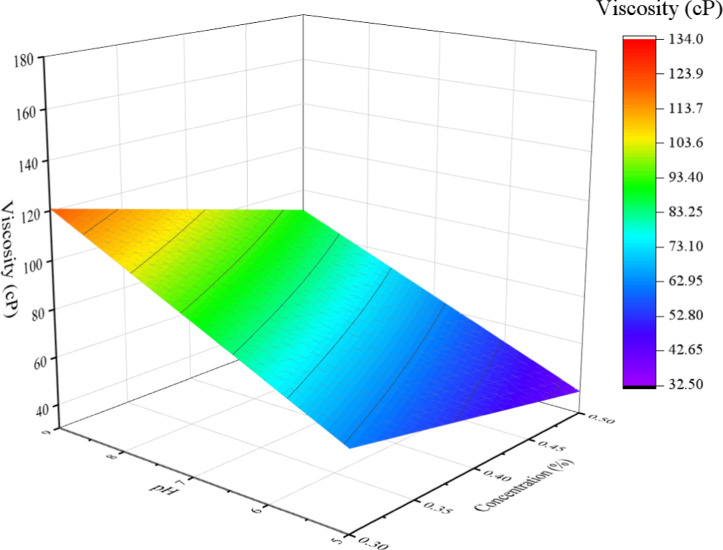
Response surface showing the effect of enzyme concentration and pH on the viscosity of WBMs at 50 °C.

A response surface methodology (RSM) approach was used to identify and quantify the factors influencing the rheological properties of the fluids after enzymatic treatment. To preserve the orthogonality of the design, the model was fitted using two levels for temperature, namely 20 and 50 °C. The surface depicted in Figure 10 was generated at a fixed temperature of 50 °C to illustrate the combined effects of enzyme concentration and pH on viscosity. The analysis of variance (ANOVA) confirmed the overall significance of the fitted model (*F* = 4425.53, *p* < 0.0001), revealing that pH and enzyme concentration were the dominant variables affecting viscosity, together with their interaction term. Temperature did not show a significant influence within the studied range and was therefore excluded from the final predictive equation. The high predictive capability of the model was confirmed by the close agreement between the Predicted *R* ^2^ (0.9976) and adjusted *R* ^2^ (0.9997) values, indicating that the model adequately represents the system’s behavior and can be reliably used for prediction.

The viscosity of the enzymatically treated drilling muds can be estimated using the following regression equation, expressed in terms of the actual factors.

3 Viscosity=66.5921−239.0729A−1.6743B+20.1704C+4.9608AB−9.5063AC−0.0742BC

where *A* represents enzyme concentration, *B* temperature, and *C* pH. This equation is suitable for predicting the viscosity within the studied range but should not be used to infer the relative importance of each factor, since the coefficients are scaled to the units of the variables and the intercept does not correspond to the center of the design space.

Overall, the results demonstrate that the combined effect of pH and enzyme concentration governs the enzymatic degradation of the polymeric matrix in drilling fluids. Operating at pH 5 maximizes enzymatic activity and promotes a marked reduction in viscosity. In contrast, when the system operates under neutral or slightly basic conditions, higher enzyme concentrations are required to achieve comparable degradation efficiency. These findings are consistent with previous reports indicating that the interaction between pH and enzyme concentration plays a critical role in enzymatic polymer degradation.^39^ While the results clearly demonstrate the potential of enzymatic treatment to reduce viscosity in BNC-based drilling fluids, this study should be considered a proof-of-concept. The experiments were conducted under controlled laboratory conditions, and no kinetic modeling of the degradation process was performed. In addition, the influence of field-relevant variables such as salinity, pressure, and the presence of drilling contaminants was not evaluated. Therefore, although the observed viscosity reduction suggests potential advantages for fluid management during cementing operations, further studies are required to validate the performance of the system under realistic operational conditions and to establish a direct link with cementing efficiency.

## Conclusions

This work demonstrates that BNC is a promising biobased additive for WBMs, capable of providing effective rheological control while contributing to more sustainable formulations. Structural characterization confirmed that BNC obtained from kombucha-derived pellicles exhibits high crystallinity, a high degree of polymerization (DPv ≈8000), and a well-developed nanofibrillar morphology, which favor the formation of robust and interconnected networks in aqueous systems.

Rheological results revealed that BNC-based WBMs exhibit higher apparent viscosities than XGD systems at equivalent bentonite concentrations, reaching values of 263 mPa·s vs 205 mPa·s (1 wt % BT) and 756 mPa·s vs 458 mPa·s (4.5 wt % BT) at 96 s^–1^. In addition, BNC systems showed a more pronounced shear-thinning behavior, reflected in lower viscosity retention values (0.31%) compared with XGD (0.61%), which facilitates flow under high shear while maintaining structure at low shear. Both polymers effectively inhibited bentonite swelling, although through different mechanisms: XGD via electrostatic interactions and BNC primarily through hydrogen bonding and steric stabilization.

Thermal aging tests at 90 °C for 24 h demonstrated that BNC-containing fluids exhibit a smaller viscosity reduction than XGD systems, retaining a high fraction of their initial viscosity and confirming their improved thermal stability and structural resilience. This behavior indicates that the fibrillar network formed by BNC is less susceptible to thermal degradation than conventional polymer chains, ensuring more stable performance under conditions relevant to drilling operations. Although slightly higher filtrate volumes were observed (≈7–15 mL), BNC-based fluids remained within the acceptable range for efficient WBMs, particularly at higher bentonite concentrations.

Furthermore, enzymatic treatment with cellulase enables controlled viscosity reduction under specific conditions (e.g., pH 5), facilitating fluid management during cementing operations. While these results demonstrate the feasibility of enzymatic degradation as a tunable strategy for postuse fluid conditioning, the present study represents a proof-of-concept under controlled laboratory conditions. Further work is required to evaluate the kinetics of degradation and to validate the performance of the system under field-relevant conditions, including the effects of salinity, pressure, and drilling contaminants.

## Abbreviations

ANOVA: analysis of variance
API: American Petroleum Institute
BNC: bacterial nanocellulose
BT: bentonite
CNC: cellulose nanocrystals
CNF: cellulose nanofibers
CUEN: cupriethylenediamine solution
DPv: viscometric degree of polymerization
FESEM: field-emission scanning electron microscopy
FTIR: Fourier transform infrared spectroscopy
PAC: polyanionic cellulose
RSM: response surface methodology
SBM: synthetic-based mud
SEM: scanning electron microscopy
SDGs: sustainable development goals
TEM: transmission electron microscopy
TGA: thermogravimetric analysis
WBM: water-based mud
XGD: xanthan gum
XRD: X-ray diffraction
